# The clock gene *PER1* plays an important role in regulating the clock gene network in human oral squamous cell carcinoma cells

**DOI:** 10.18632/oncotarget.11844

**Published:** 2016-09-02

**Authors:** Qin Zhao, Gang Zheng, Kai Yang, Yi-ran Ao, Xiao-li Su, Yu Li, Xiao-qiang Lv

**Affiliations:** ^1^ Department of Oral and Maxillofacial Surgery, The First Affiliated Hospital of Chongqing Medical University, Yuzhong District, Chongqing 400016, China; ^2^ Chongqing Traditional Chinese Medicine Hospital, Chongqing 400021, China; ^3^ Department of Pathology, Chongqing Medical University, Chongqing 400016, China

**Keywords:** circadian clock, oral cancer, PER1, gene

## Abstract

The various clock genes in normal cells, through their interaction, establish a number of positive and negative feedback loops that compose a network structure. These genes play an important role in regulating normal physiological activities. The expression of clock gene *PER1* is decreased in many types of cancer. *PER1* is highly correlated with the initiation and progression of cancer by regulating numerous downstream genes. However, it is still unclear whether the lower expression of *PER1* in cancer can influence the expression of other clock genes in the clock gene network. In this study, we used short hairpin RNA interference to knock down *PER1* effectively in SCC15 human oral squamous cell carcinoma cells. These cancer cells later were subcutaneously injected into the back of nude mice. We discovered that after *PER1* knockdown, apoptosis was decreased and cell proliferation and *in vivo* tumor formation were enhanced. Quantitative real-time PCR result indicated that *in vitro* and *in vivo* cancer cells after *PER1* knockdown, *PER2, DEC1, DEC2, CRY1, CRY2 and NPAS2* were significantly down-regulated at the mRNA level, while *PER3*, *TIM*, *RORα* and *REV-ERBα* were significantly up-regulated. It prompts that the role of *PER1* in carcinogenesis is exerted not only by regulating downstream genes, but also through the synergistic dysregulation of many other clock genes in the clock gene network.

## INTRODUCTION

Circadian clock, is present in the human body, has important effects on the tightly organized regulation of various complex physiological processes [1, 2]. The clock genes are the core structure of circadian clock. To date, 14 clock genes have been identified: *CLOCK, BMAL1, PER1, PER2, PER3, DEC1, DEC2, CRY1, CRY2, TIM, CKIε, RORα*, NPAS2 and *REV-ERBα* [2–10], which are nearly ubiquitously expressed in humans [11, 12]. The clock genes interact and form networks through a set of positive and negative feedback loops at the transcriptional and translational levels. These genes form the clock gene networks through interaction [3–8]. About 2-10% of mammalian genes are regulated genome-wide by the products of these clock genes, which are known as clock-controlled genes (CCGs) [3, 13, 14]. As Zhang et al. reported 43% of all protein coding genes are CCGs [15]. Different clock genes can affect cellular activities by regulating the expression of several downstream CCGs [3]. The abnormal expression of clock genes is significant causes that lead to the occurrence and progression of many diseases, including cancer, cardiovascular disease, diabetes and depression [3, 9, 16].

*Period 1 (PER1)* is an important circadian clock gene [4, 5]. Recent reports have indicated that *PER1* expression is decreased in a series of solid carcinomas, such as head-neck carcinoma, prostatic cancer, breast cancer, colorectal cancer, and endometrial cancer [17–20]. *PER1* can regulate downstream cell cycle genes. In addition, reduced mRNA expression of *PER1* can lead to an imbalance between cell proliferation and apoptosis, further promoting malignant cell transformation [9, 10, 18, 21–23]. We previously demonstrated that mRNA and protein expression of *PER1* are remarkably reduced in oral squamous cell carcinoma (OSCC) compared to para-carcinoma tissue [17]. We also illustrated that *PER1* knockdown in OSCC cells SCC15 results in altered expression of numerous downstream cell cycle genes and cancer-related genes, which enhanced proliferation and metastasis of cancer cell [24]. Above all, it is universally accepted that decreased *PER1* expression in cancer closely correlates with tumor occurrence and progression by regulating downstream cell cycle genes and cancer-related genes, including *Cyclin B1, Cyclin D, Cyclin E, WEE-1, C-MYC, KI-67, MDM2,* and *p53* [12, 18, 24]. *PER1* gene is one of the most important components in the clock gene network. However, it is still unclear whether reduced *PER1* expression in carcinoma cells can affect the normal expression of other clock genes in the inherent network. Here, we used short hairpin RNA (shRNA) interference to effectively knockdown *PER1* in SCC15 human OSCC cells. We demonstrated that *in vitro* and *in vivo* cancer cells after *PER1* knockdown, *PER2, DEC1, DEC2, CRY1, CRY2 and NPAS2* were significantly down-regulated at the mRNA level, while *PER3*, *TIM*, *RORα* and *REV-ERBα* were significantly up-regulated. In addition, we determined that apoptosis was decreased, whereas cell proliferation and tumor formation were enhanced, after *PER1* knockdown *in vitro* and *in vivo*. Our findings prompt that the effect of *PER1* on tumor occurrence and progression is not only achieved by regulating downstream CCGs, but also through the synergistic modulation of other clock genes in the network.

## RESULTS

### Construction and sequencing of lentivirus shRNA plasmids

DNA sequencing results of the lentiviral PER-shRNA-I~III plasmids are reported in Supplementary Figure S1 and Supplementary Table S1. The plasmid sequences exactly matched the oligonucleotide interference target sequences of positive-sense strands of PER-shRNA-I~III, indicating that three shRNAs targeting *PER1* were successfully constructed.

### Expression of PER1 mRNA and protein in tumor cells

**Figure 1:**
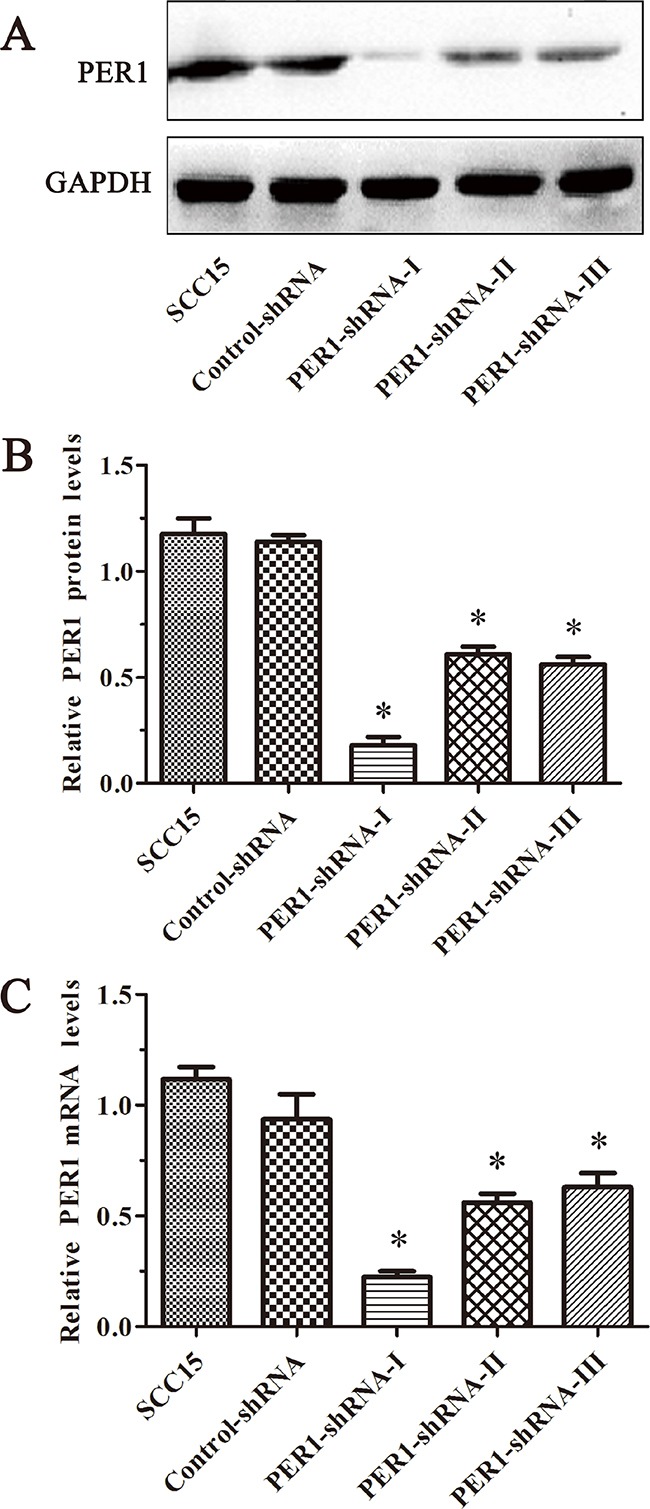
PER1 is most efficiently knocked down in the PER1-shRNA-I group among five groups of SCC15 cells **A.** Levels of PER1 protein in the SCC15, Control-shRNA and PER1-shRNA-I-III groups. **B.** Levels of PER1 protein were significantly reduced in SCC15 cells transfected with PER1-shRNA-I. **C.** Expression of PER1 mRNA was significantly down-regulated in SCC15 cells transfected with PER1-shRNA-I. Data are presented as the mean ± SD. Significant differences between multiple groups were evaluated using ANOVA; differences between two groups were evaluated using the LSD test. *P<0.05 was considered statistically significant.

qRT-PCR analysis showed that in the three PER1-shRNA-I~III groups, Control-shRNA group, and untreated SCC15 cells, mRNA expression of PER1 normalized to the level of β-actin mRNA were 0.22±0.05, 0.56±0.07, 0.63±0.11, 0.94±0.20 and 1.12±0.10, respectively. Western blot data indicated that the relative level of PER1 protein normalized to the level of GAPDH protein was 0.18±0.07, 0.61±0.06, 0.56±0.06, 1.14±0.05 and 1.18±0.13, respectively. The expression of PER1-shRNA-I was significantly reduced both at the mRNA and protein levels, compared to the other groups (*P<0.05*) (Figure 1). This demonstrates that *PER1* was most effectively knocked down in PER1-shRNA-I group. Therefore, we chose to use this shRNA for the following experiments.

### Proliferation and apoptosis of SCC15 cells *in vitro* after *PER1* knockdown

**Figure 2:**
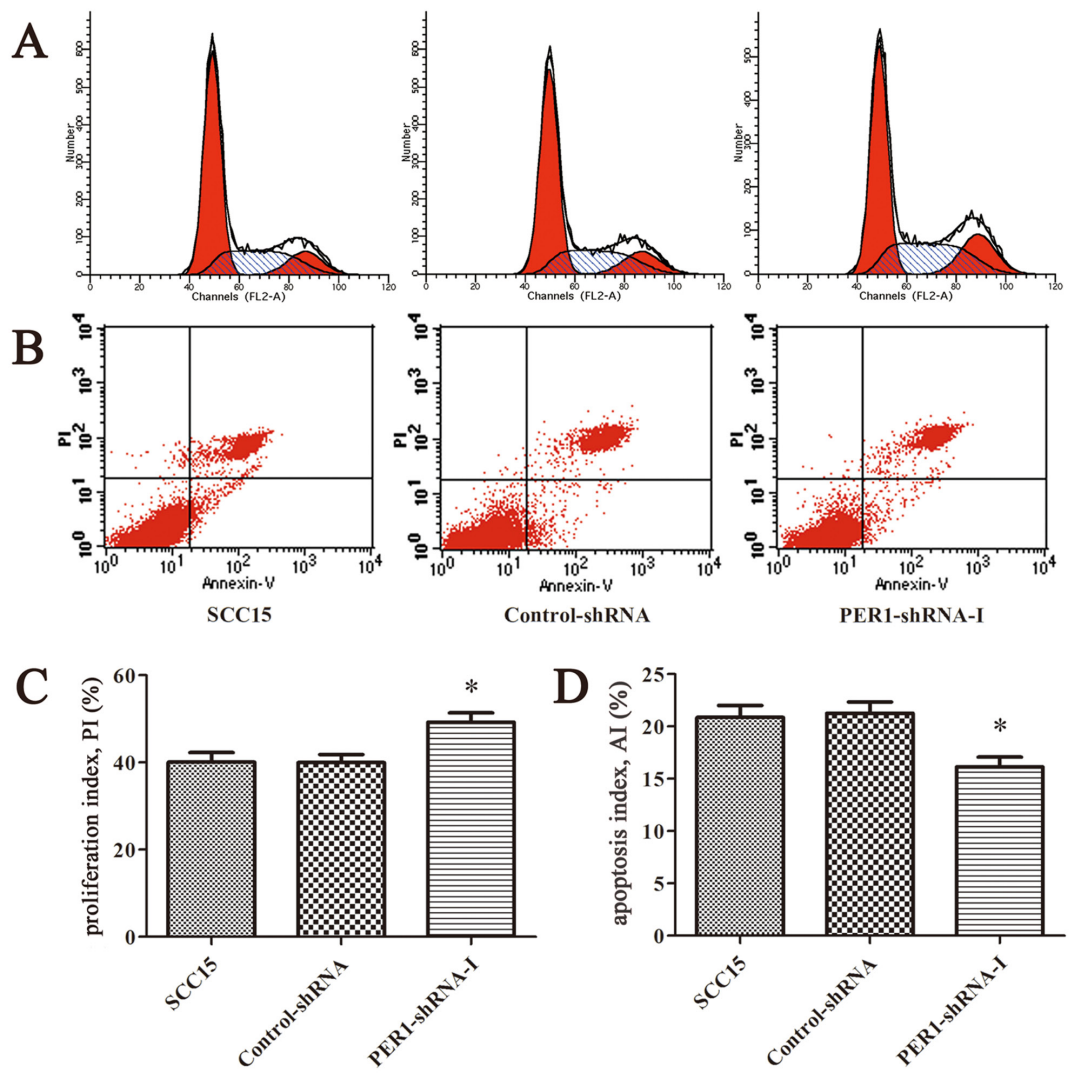
*PER1* knockdown enhances proliferation and reduces apoptosis of SCC15 cells *in vitro* **A.** Representative flow cytometry profiles of proliferation among cells in the SCC15, Control-shRNA and PER1-shRNA-I groups. **B.** Representative flow cytometry profiles of apoptosis among cells in the SCC15, Control-shRNA and PER1-shRNA-I groups. **C.** The proliferation index was significantly increased in SCC15 cells transfected with PER1-shRNA-I *in vitro*. **D.** The apoptosis index was significantly reduced in SCC15 cells transfected with PER1-shRNA-I *in vitro*. Data are presented as the mean ± SD. Differences between multiple groups were evaluated using ANOVA; differences between two groups were evaluated using the LSD test. *P<0.05 was considered statistically significant.

Flow cytometry analysis indicated that the cell proliferation index of the cells expressing PER1-shRNA-I (49.21±3.75%) was significantly higher than for cells expressing Control-shRNA (39.99±3.19%) or untreated SCC15 cells (40.09±3.82%) (*P<0.05*). The cell apoptosis index of the cells expressing PER1-shRNA-I (16.61±1.37%) was significantly lower than for cells expressing Control-shRNA (21.26±1.90%) or untreated SCC15 cells (20.86±2.00%) (*P<0.05*). In contrast, no difference was noted between the Control-shRNA group and the SCC15 group either in the proliferation index or in the apoptosis index (*P>0.05*) (Figure 2).

### mRNA expression levels of clock genes in SCC15 cells after *PER1* knockdown *in vitro*

**Figure 3:**
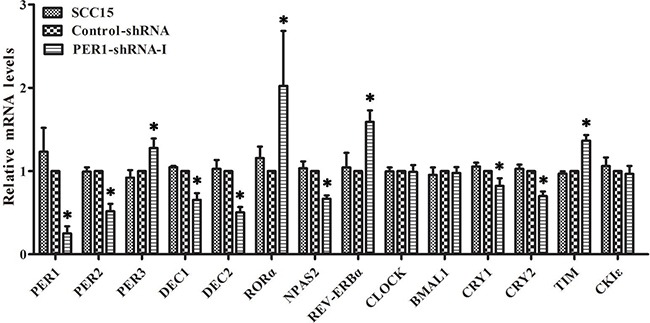
Levels of mRNA expression of clock genes in SCC15 cells after *PER1* knockdown *in vitro* mRNA expression of PER3, TIM, RORα and REV-ERBα was significantly up-regulated in the PER1-shRNA-I group as compared to the Control-shRNA and SCC15 groups, while mRNA expression of PER2, DEC1, DEC2, CRY1, CRY2 and NPAS2 was significantly down-regulated. There was no notable difference between the Control-shRNA and SCC15 groups. In addition, there was no difference in mRNA expression of CLOCK, BMAL1 and CKIε among the three groups. Data are presented as the mean ± SD. Differences between multiple groups were evaluated using ANOVA; differences between two groups were evaluated using the LSD test. *P<0.05 was considered statistically significant.

qRT-PCR analysis demonstrated that the mRNA expression of PER3, TIM, RORα and REV-ERBα was significantly up-regulated in the PER1-shRNA-I group when compared to the Control-shRNA and SCC15 groups (*P<0.05*). In contrast, the mRNA expression of PER2, DEC1, DEC2, CRY1, CRY2 and NPAS2 was significantly down-regulated (*P<0.05*). There was no notable difference between the Control-shRNA and SCC15 groups (*P>0.05*). In addition, there was no difference in the mRNA expression of CLOCK, BMAL1 and CKIε among the three groups (*P>0.05*) (Table 1 and Figure 3).

**Table 1:** Levels of mRNA expression of clock genes in the PER1-shRNA-I, Control-shRNA and SCC15 groups *in vitro* (mean±SD)

Gene	PER1-shRNA-I	Control-shRNA	SCC15	F	*P*	*P_1_*	*P_2_*	*P_3_*
*PER1*	0.25±0.09	1.00	1.23±0.29	26.09	0.001	0.002	0.000	0.156
*PER2*	0.52±0.09	1.00	0.99±0.05	64.24	0.000	0.000	0.000	0.871
*PER3*	1.28±0.11	1.00	0.92±0.09	14.64	0.005	0.007	0.002	0.259
*DEC1*	0.65±0.08	1.00	1.05±0.02	56.02	0.000	0.000	0.000	0.293
*DEC2*	0.51±0.06	1.00	1.03±0.11	50.21	0.000	0.000	0.000	0.665
*CRY1*	0.82±0.09	1.00	1.05±0.05	12.34	0.007	0.011	0.003	0.317
*CRY2*	0.70±0.05	1.00	1.03±0.05	53.08	0.000	0.000	0.000	0.476
*TIM*	1.36±0.07	1.00	0.97±0.03	87.12	0.000	0.000	0.000	0.401
*RORα*	2.02±0.66	1.00	1.16±0.14	6.05	0.036	0.018	0.034	0.639
*NPAS2*	0.67±0.04	1.00	1.03±0.08	44.36	0.000	0.000	0.000	0.469
*REV-ERBα*	1.59±0.14	1.00	1.04±0.18	19.55	0.002	0.001	0.002	0.695
*CLOCK*	0.99±0.08	1.00	1.00±0.05	0.04	0.964	0.801	0.856	0.943
*BMAL1*	0.98±0.07	1.00	0.95±0.09	0.37	0.705	0.693	0.670	0.422
*CKIε*	0.97±0.10	1.00	1.06±0.10	1.00	0.421	0.630	0.212	0.408

Notes: *P* values reflect differences in each gene expression among the three groups analyzed using one-way ANOVA.

*P_1_*, *P_2_*, and *P_3_* respectively reflect the intergroup differences between the PER1-shRNA-I and Control-shRNA groups, the PER1-shRNA-I and SCC15 groups, and the Control-shRNA and SCC15 groups analyzed using the LSD test after one-way ANOVA. Values of *P<0.05* were considered statistically significant.

### Tumorigenesis assay in nude mice *in vivo*

**Figure 4:**
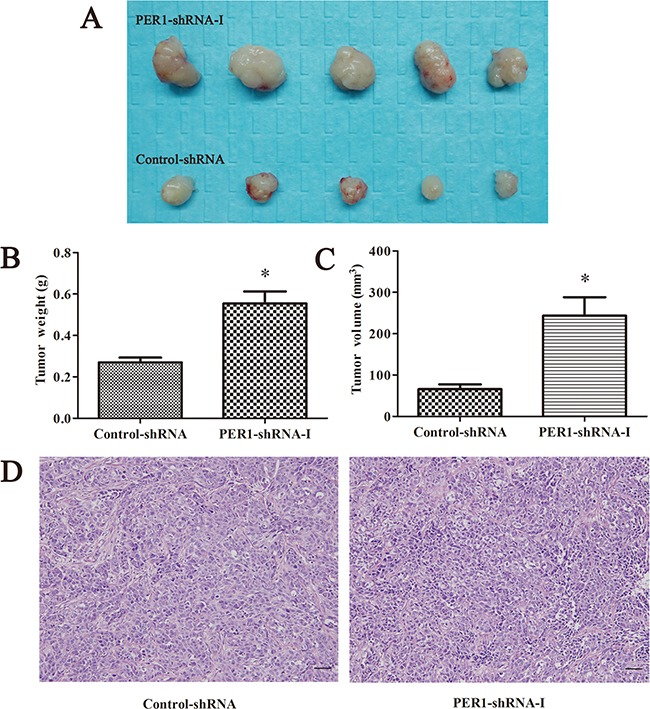
*PER1* knockdown enhances the tumorigenic capacity of SCC15 cells *in vivo* **A.** The condition of tumorigenesis *in vivo* including PER1-shRNA-I and Control-shRNA group cells. **B** and **C**. Average weight and volume of transplanted tumors from nude mice were significantly increased after *PER1* knockdown. **D.** HE staining of tumor tissue slices in the PER1-shRNA-I and Control-shRNA group were observed under an optical microscope (200×). Data are presented as the mean ± SD, Student's t-test was used to analyze the significance of differences. *P<0.05 was considered statistically significant.

All of the ten nude mice survived and presented tumor growth. The tumor weights in the PER1-shRNA-I and Control-shRNA groups were, respectively, (0.55±0.13) g and (0.27±0.05) g. The tumor volumes in the PER1-shRNA-I and Control-shRNA groups were, respectively, (0.24±0.10) cm^3^ and (0.07±0.03) cm^3^. The average weight and volume of transplanted tumors from nude mice in the PER1-shRNA-I group was significantly increased compared to the Control-shRNA group (*P<0.05*). They appeared as squamous cell carcinoma by HE staining of tumor slices observed under an optical microscope (200×) (Figure 4).

### Proliferation and apoptosis of tumor cells after *PER1* knockdown *in vivo*

**Figure 5:**
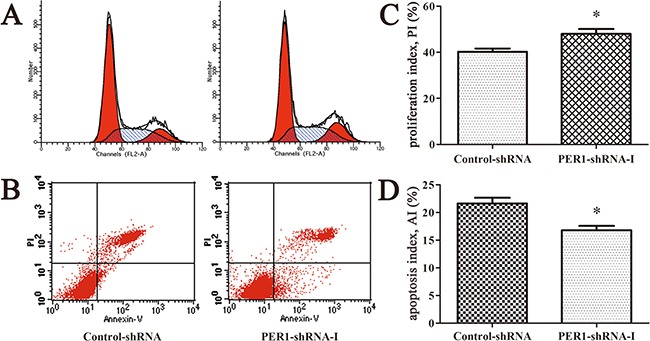
*PER1* knockdown enhances proliferation and reduces apoptosis of SCC15 cells *in vivo* **A.** Representative flow cytometry profiles of proliferation among tumor tissues in the Control-shRNA and PER1-shRNA-I groups. **B.** Representative flow cytometry profiles of apoptosis among tumor tissue in the Control-shRNA and PER1-shRNA-I groups. **C.** The proliferation index was significantly increased in SCC15 cells transfected with PER1-shRNA-I *in vivo*. **D.** The apoptosis index was significantly reduced in SCC15 cells transfected with PER1-shRNA-I *in vivo*. Data are presented as the mean ± SD. Student's t-test was used to analyze the significance of differences. *P<0.05 was considered statistically significant.

The *in vivo* cell proliferation index of cells expressing PER1-shRNA-I (47.96±3.69%) was significantly higher than for cells expressing Control-shRNA (40.22±2.45%) (*P <0.05*). The *in vivo* cell apoptosis index of cells expressing PER1-shRNA-I (16.78±1.39%) was significantly lower than for cells expressing Control-shRNA (21.64±1.75%) (*P<0.05*) (Figure 5). These results indicate that the alterations in proliferation and apoptosis of SCC15 cells *in vivo* are in accordance with the *in vitro* data observed after *PER1* knockdown.

### mRNA expression of clock genes in cancer cells after *PER1* knockdown *in vivo*

**Figure 6:**
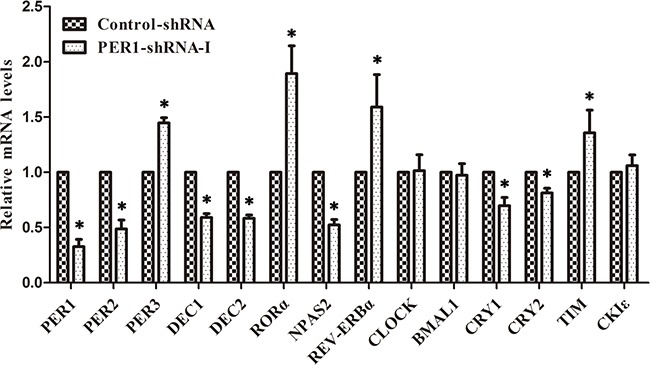
Levels of mRNA expression of clock genes after *PER1* knockdown *in vivo* (mean ± SD) mRNA expression of PER3, TIM, RORα and REV-ERBα was significantly up-regulated in the PER1-shRNA-I group as compared to the Control-shRNA, while mRNA expression of PER2, DEC1, DEC2, CRY1, CRY2 and NPAS2 was significantly down-regulated. There was no difference in mRNA expression of CLOCK, BMAL1 and CKIε among the two groups. Data are presented as the mean ± SD. Student's t-test was used to analyze the significance of differences. *P<0.05 was considered statistically significant.

qRT-PCR analysis showed that the mRNA expression of PER1, PER2, DEC1, DEC2, CRY1, CRY2 and NPAS2 was significantly down-regulated *in vivo* in the PER-shRNA-I group as compared to the Control-shRNA group tumors (*P<0.05*), while mRNA expression of PER3, TIM, RORα and REV-ERBα was significantly up-regulated (*P<0.05*), and mRNA expression of CLOCK, BMAL1 and CKIε had no obvious change among the two groups (*P >0.05*) (Table 2 and Figure 6). These observations suggest that the mRNA expression patterns of clock genes in SCC15 cancer cells are similar *in vitro* and *in vivo*.

**Table 2:** Levels of mRNA expression of clock genes in the PER1-shRNA-I and Control-shRNA tumor cell groups *in vivo* (mean±SD)

Gene	PER1-shRNA-I	Control-shRNA	F	*P*
*PER1*	0.33±0.07	1.00	312.75	0.000
*PER2*	0.49±0.08	1.00	117.74	0.000
*PER3*	1.45±0.05	1.00	268.64	0.000
*DEC1*	0.59±0.04	1.00	392.11	0.000
*DEC2*	0.58±0.03	1.00	654.31	0.000
*CRY1*	0.70±0.07	1.00	51.68	0.002
*CRY2*	0.81±0.04	1.00	55.25	0.002
*TIM*	1.36±0.21	1.00	9.07	0.040
*RORα*	1.89±0.25	1.00	37.99	0.004
*NPAS2*	0.53±0.05	1.00	288.10	0.000
*REV-ERBα*	1.59±0.30	1.00	12.10	0.025
*CLOCK*	1.02±0.14	1.00	0.04	0.861
*BMAL1*	0.97±0.10	1.00	0.23	0.657
*CKIε*	1.06±0.10	1.00	1.11	0.352

Notes: *P* values reflect differences in each gene expression among the PER1-shRNA-I and Control-shRNA groups analyzed using student's t-test. Values of *P<0.05* were considered statistically significant.

## DISCUSSION

Previous studies have demonstrated that the expression of *PER1* is decreased in many types of cancer. Further, *PER1* is able to regulate numerous downstream cell cycle genes and cancer-related genes that act as CCGs, thereby it has a close relationship with tumor occurrence and progression [12, 17–20, 24]. *PER1* is an important circadian clock gene [4, 5], this study demonstrates that *PER1* also has an important role in regulating the clock gene network.

Recent studies have demonstrated that in normal cells the clock genes mainly create networks through three feedback loops at the transcriptional and translational levels. Positive feedback factors of these three loops are CLOCK/BMAL1 heterodimers, which serve as transcription factors to activate the transcription of *PERs, CRYs, DECs, REV-ERBα* and *RORα* genes and promote their expression, thus generating the positive feedback pathway. In contrast, PERs and CRYs proteins form heterodimers and translocate into the nucleus to get feedback on inhibiting and reducing the transcriptional activation of CLOCK/BMAL1 through combining with it, thus generating the first negative feedback pathway. In the next place, DEC1 and DEC2 form hetero- or homodimers, as the transcription factor, which translocate into the nucleus to reduce the transcriptional activation of CLOCK/BMAL1 heterodimers competitively, and generate the second negative feedback pathway. Finally, REV-ERBα and RORα proteins translocate into the nucleus to repress and promote *BMAL1* expression as the transcription factor respectively, thus representing the third negative feedback pathway [4–8]. In our research, we found no marked alterations in the mRNA expression of CLOCK and BMAL1 after *PER1* knockdown in SCC15 cells, suggesting that *CLOCK* and *BMAL1* are not subject to *PER1* regulation at the transcriptional level. Lee et al. [25] reported that CLOCK/BMAL1 remain steady during circadian feedback loops in mammalian liver normal cells. As a transcriptional regulation factor, CLOCK/BMAL1 plays the positive regulating role by nuclear translocation. Hence, we speculate that the lower expression of *PER1 in cancer* cells can influence the activation and the intracellular distribution of CLOCK/BMAL1, rather than the alteration in expression level.

Based on the above feedback loops in normal cells, when the expression of *PER1* decreased, reducing the inhibitory effect of the positive transcriptional regulator CLOCK/BMAL1 heterodimer, as a result, increasing the transcriptional activation of CLOCK/BMAL1 and the expression of negatively regulated genes, including *PER2, PER3, CRY1, CRY2, DEC1, DEC2, REV-ERBα* and *RORα*. Zheng et al. reported that in *PER1* mutant mice, PER2 protein expression was increased [11]. Further, Jacob and colleagues [26] demonstrated that *PER1* knockdown in mouse liver and endothelial normal cells resulted in the up-regulation of *CRY2* at the mRNA and protein levels. However, our *in vitro* and *in vivo* experiments demonstrated that after *PER1* knockdown in SCC15 cells, only the expression of *PER3, RORα* and *REV-ERBα* increased among the negatively regulated genes, in agreement with previously identified mechanisms of the positive and negative circadian feedback regulation in normal cells. The mRNA expression of other negatively regulated genes, including *PER2, CRY1, CRY2, DEC1* an*d DEC2*, was markedly decreased, in contrast with the typical responses observed for the positive and negative circadian feedback regulation in normal cells. We speculated the reason may be that previous experiments were all performed in normal cells, while our research was performed in cancer cells. We might assume that the regulatory functions of the positive and negative circadian feedback loop network in cancer cells may be distinct from that in normal cells, or there might exist additional complementary regulatory processes in cancer cells. Moreover, there may exist other mechanisms in cancer cells to regulate the expression of clock genes, leading to the abnormal feedback loops of clock genes. For instance, Kuo and Clark el. proved that promoters of these clock genes (*PER1, PER2, PER3, CRY1* and *CRY2*) were methylated in a variety of cancer cells, which resulted in their decreased expression [27, 28]. The above problems need to be further in-depth study.

We also found that after *PER1* knockdown in SCC15 cells, mRNA expression of TIM and NPAS2 were greatly increased or decreased, respectively. *TIM* has not been included in the circadian feedback loops of normal cells so far [4–8]. We determined that the mRNA expression of TIM was increased after *PER1* knockdown in cancer cells, indicating that *TIM* may either serve as a factor in the circadian feedback loops of cancer cells. NPAS2 is a paralog of CLOCK that can dimerize with BMAL1 to form NPAS2/BMAL1 heterodimers, which have a similar function to CLOCK/BMAL1 [29], and it appears to have a compensatory effect on CLOCK/BMAL1 [30]. In our research, mRNA expression of NPAS2 was robustly decreased after *PER1* knockdown, indicating that the compensatory effect on CLOCK/BMAL1 was weakened due to *PER1* knockdown in cancer cells.

At present, it is recognized that altered *PER1* expression could result in altered expression of downstream cell cycle genes and cancer-related genes, thus leading to the occurrence of tumors [12, 18, 24]. Our study clarifies that *PER1* has an important role in regulating the clock gene network, and for the first time finds that the regulation function has a large difference between normal cells and cancer cells. This study demonstrates that the down-regulation of *PER1 in cancer cells can result in the robust up-regulation of PER3, TIM, RORα* and *REV-ERBα* transcripts, while the expression of *PER2, DEC1, DEC2, CRY1, CRY2* and *NPAS2* is prominently down-regulated *in vitro* and *in vivo*. We also determined that apoptosis was decreased and cell proliferation and tumor formation were enhanced after *PER1* knockdown *in vitro* and *in vivo*. Our research prompts that the role of *PER1* on tumor occurrence and progression is not only exerted by regulating downstream CCGs, but also by simultaneously modulating the expression of many other clock genes in the clock gene network. However, our research only concentrated on the transcriptional level. Future studies will need to concentrate on the translational and post-translational levels, to further illustrate the molecular function and the regulatory effects in the clock gene network and the tumor suppression mechanisms of *PER1*, providing new and effective molecular targets for the treatment of cancers.

## MATERIALS AND METHODS

### Construction and identification of lentivirus shRNA plasmids

shRNA target sequences for *PER1* were selected by contrast and screening principles [31] on the basis of the GenBank mRNA sequence encoding hPER1 (NM_002616.2), (PER1-I, CAGCACCACT AAGCGTAAATG; PER1-II, CCAGCACCACTAAGCGT AAAT; PER1-III, CCATGGACATGTCCACCTATA). Then, using the design principles for RNA interference [31], three pairs of shRNA plasmids targeting *PER1* (PER1-shRNA-I, PER1-shRNA-II and PER1-shRNA-III) were designed (Table 3). Restriction enzyme digestion with AgeI and EcoRI (NEB, Ipswich, Massachusetts, USA) was used to linearize the vector plasmid PLKO.1 after gel electrophoresis. Next, we incubated the PER1-shRNA-I~III annealing primers with T4 DNA Ligase, to construct the lentiviral PER1-shRNA-I~III plasmids. The scramble shRNA 5'-CCTAAGGTTAAGTCGCCCTCGCTCGAGCGAGGGCGACTTAACCTTA GG-3' (Sigma-Aldrich, St. Louis, MO, USA), which had no predicted interference effects on any genes, served as the control (Control-shRNA). These vectors were then transformed into freshly prepared Escherichia coli DH5α cells (Sangon Biotech, Shanghai, China). Bacterial colonies were then selected in LB medium with Amino nucleoside antibiotic and cultured at 37°C overnight. Plasmids were extracted using a QIAGEN Plasmid Midi Kit (Qiagen, Germany), and the amplification products produced by PCR were analyzed by DNA sequencing using the Chromas v2.4 program (Technelysium, Australia).

**Table 3:** Sequences of PER1-shRNAs

Group	Sense strand	Antisense Strand
PER1-shRNA-I	5′-CCGGCAGCACCACTAAGCGTAAATG CTCGAGCATTTACGCTTAGTGGTGCTGT TTTTG-3′	5′-AATTCAAAAACAGCACCACTAAGCGTA AATGCTCGAGCATTTACGCTTAGTGGTGC TG-3′
PER1-shRNA-II	5′-CCGGCCAGCACCACTAAGCGTAAATCT CGAGATTTACGCTTAGTGGTGCTGGTTTT TG-3′	5′-AATTCAAAAACCAGCACCACTAAGCGT AAATCTCGAGATTTACGCTTAGTGGTGCT GG-3′
PER1-shRNA-III	5′-CCGGCCATGGACATGTCCACCTATACT CGAGTATAGGTGGACATGTCCATGGTTTT TG-3'	5′-AATTCAAAAACCATGGACATGTCCACCTA TACTCGAGTATAGGTGGACATGTCCATGG-3'

### Lentivirus shRNA plasmid packing

For transfection, 1 ml DMEM, 10 μl plasmid, 10 μl Lenti-HG Mix and 60 μl HG transgene reagent (Qiagen, Germany) were mixed together at room temperature for 20 min, then added to 70-80% confluent 293T cells (Life Sciences Institute of Chongqing Medical University, China), according to the manufacturer's protocol. Based on a QIAGEN Plasmid Packaging Midi Kit (Qiagen, Germany), titrate 120 μl 100× Enhancing buffer after 12 h. After incubation for 48 h, 293T cells supernatant was filtered using 0.45 μm cellulose acetate filters. Four different lentiviruses were collected and stored at −80°C until further use.

### Cell transfection

During logarithmic growth, SCC15 cells (Life Sciences Institute of Chongqing Medical University, China) were seeded into six well plates (NO.1~6) with 1.5 ml of DMEM/F12 supplemented with 10% fetal bovine serum (FBS). 500 μl PER1-shRNA-I, PER1-shRNA-II, PER1-shRNA-III and Control-shRNA, each containing 2.5 μl Polybrene, were added to the first 4 wells. 500 μl of serum-free DMEM/F12 were added to the remaining two wells. After 24 h incubation at 37°C, in the presence of 5% CO2, fresh medium was added to the plate. The first five wells received DMEM/F12 containing 2 μg/ml puromycin, while the sixth well received serum-free DMEM/F12. Stably transfected cells were obtained after 7 days, and the culture medium was replaced every day. The transfectants were divided into five groups: three experimental groups expressing PER1-shRNA-I~III, a Control-shRNA group expressing scramble shRNA, and an untreated SCC15 cell group (blank control group).

### Western blot analysis

Exponential phase cells were lysed in RIPA buffer [50 mmol/L Tris- HCl (pH 7.4), 150 mmol/L NaCl, 0.5% NP-40] for 30 min at 0°C. Cell lysates were collected using a cell scraper and centrifuged for 5 min (12,000 rpm, 4°C), and then the supernatants were transferred to new tubes. Protein concentration was quantified using a BCA Protein Assay Kit according to the manufacturer's instructions (Beyotime, Jiangsu, China). The supernatants (50 μg protein) were subjected to SDS-PAGE, after which the proteins were transferred to polyvinylidene fluoride (PVDF) membranes (Millipore, USA), which were then blocked with 5% non-fat dried milk for 1 h. The membranes were then probed with rabbit polyclonal anti-hPER1 antibody (1:1000, Genetex, USA) and mouse monoclonal anti-hGAPDH antibody (1:3000, Zhongshan Golden-Bridge Biotechnology, China) for 2 h at room temperature. The membranes were washed three times in TBS, and then incubated with horseradish peroxidase-conjugated goat anti-rabbit IgG (1:5000, Zhongshan Golden-Bridge Biotechnology, China) at 37°C for 2 h. The precipitated proteins were washed three times in TBS, and an ECL-advance Western Blot Detection System (Bio-Rad, California, USA) was used for detection and photography. The assays were done in triplicate.

### Flow cytometry assay

Cells in logarithmic growth phase were harvested by trypsinization (0.25%) and centrifuged for 5 min (1000 rpm, 4°C), after which the supernatant was discarded and the cell pellets were washed twice with PBS and resuspended in PBS at a density of 1×10^6^cells/ml. (1) Detection of cell proliferation: cell suspensions (1 ml) were centrifuged for 5 min (800 rpm, 4°C), and the supernatants were discarded. Then, 70% ethyl alcohol (−20°C, 1ml) was added to the cell pellets, repeatedly mixed, and incubated overnight at 4°C. The cell suspensions were washed twice with PBS and resuspended in PBS (1 ml). 1 ml of propidium iodide solution was added to the cell suspensions and incubated for 30 min at 4°C in the dark. Proliferation was analyzed using FACSVantage flow cytometry (BD, New Jersey, USA), and the ModFit LT 2.0 program (Verity Software House, Topsham, Maine, USA) was used for fitting analysis. The following formula was used to calculate the tumor cells proliferation index (PI): PI=(S+G2/M)/(G0/G1+S+G2/M) × 100%. (2) Detection of cell apoptosis: cell suspensions (1 ml) were fixed in 70% ethyl alcohol for 2 h, and then resuspended in Binding Buffer (0.5 ml). The cell suspensions were incubated with 200 μl of AnnexinV-FITC (Beyotime, Jiangsu, China) reagent for 15 min in the dark, and then stained with 1 ml of propidium iodide solution for 2 min at room temperature in the dark. Apoptosis was analyzed using FACSVantage flow cytometry. The following formula was used to calculate the tumor cells apoptotic index (AI): AI = (number of apoptotic cells/total number of cells) × 100%. Each experiment was carried out in triplicate.

### Quantitative real-time PCR assay (qRT-PCR)

Total RNA was extracted from cells using RNAiso Plus (Takara, Kyoto, Japan). RNA concentration and quality were determined using a UV/Visible spectrophotometer (Amersham Biosciences, Goteborg, Sweden) to measure absorbance at 260 nm and 280 nm. The RNAs were reverse transcribed by cDNA synthesis at 37°C for 15 min and 85°C for 5s. The qRT-PCR primers for *CLOCK, BMAL1, PER1, PER2, PER3, DEC1, DEC2, CRY1, CRY2, TIM, CKIε, RORα,* NPAS2 and *REV-ERBα* were designed using Oligo7.0 software, and are listed in Table 4. β-actin served as a normalization control. The reaction mixture for qPCR contained 12.5 μl of 2× SYBR Premix Ex TaqTMII, 1 μl of 0.4 μM forward and reverse primers, 2μl of 50 ng/μl cDNA template and 8.5 μl double distilled H_2_O in a total volume of 25 μl. qPCR was performed using a C-1000TM Thermal Cycler (Bio-Rad, California, USA). The PCR protocol entailed pre-denaturation at 95°C for 1.5 min, and then amplification for 40 cycles, including denaturation for 10s at 95°C, and annealing extension for 30s at 60°C. The cycle threshold (Ct) values were determined and normalized against the expression of β-actin in each sample. The data were analyzed using the 2^−ΔΔCt^ method. The assays were done in triplicate.

**Table 4:** Primer sequences for clock genes used for quantitative real-time PCR

Gene	Forward primer sequence	Reverse primer sequence
*PER1*	5′-CTGCTACAGGCACGTTCAAG-3′	5′-CTCAGGGACCAAGGCTAGTG-3′
*PER2*	5′-TTGGACAGCGTCATCAGGTA-3′	5′-TCCGCTTATCACTGGACCTT-3′
*PER3*	5′-GCAGGTCTATGCCAGTGTGA-3′	5′-ACCACCACCATTCGGTTCT-3′
*CLOCK*	5′-CAGCCAGTGATGTCTCAAGC-3′	5′-ATGCGTGTCCGTTGTTCC-3′
*BMAL1*	5′-TGCCACCAATCCATACACAG-3′	5′-TTCCCTCGGTCACATCCTAC-3′
*DEC1*	5′-CAGCTTTCGGATGATGAAGG-3′	5′-GCTGAAGGTGGGATCAGGTA-3′
*DEC2*	5′-GGGACCAACTGCTTCACACT-3′	5′-TAATCTGTGGGACGGTAGGC-3′
*CRY1*	5′-TGTGATTCGTGGACAACCAG-3′	5′-TAGCTGCGTCTCGTTCCTTT-3′
*CRY2*	5′-AGGAGAACCACGACGAGA-3′	5′-TCCGCTTCACCTTTTTATAC-3′
*CKIε*	5′-TGAGTATGAGGCTGCACAGG-3′	5′-CTTCCCGAGATGGTCAAATG-3′
*TIM*	5′-GATAGAGGCCCATTCCTGCAT-3′	5′-GAAGGGCTGGGGAACTTAGAC-3′
*NPAS2*	5′-AACCTCGGCAGCACTTTAAC-3′	5′-GGTTCTGACATGGCTGTGTG-3′
*RORα*	5′-CTATCCCTCCAAGGCACAAG-3′	5′-AACACAAGACTGACGAGCACA-3′
*REV-ERBα*	5′-ACAGAATCGAACTCTGCACTTCT-3′	5′-GGGGAGGGAGGCAGGTATT-3′
*β-actin*	5′-AGCGAGCATCCCCCAAAGTT-3′	5′-GGGCACGAAGGCTCATCATT-3′

### *In vivo* tumorigenicity assay

Ten specific pathogen-free (SPF) BALB/c nu/nu nude mice (females, 4-6 weeks old, 18-22 g) were purchased from the Experimental Animal Center of the Chongqing Medical University, and evenly divided into two groups of five mice each: PER1-shRNA-I (experimental group) and Control-shRNA (control group). During the logarithmic growth, PER1-shRNA-I and Control-shRNA cells were digested with 0.25% trypsin, centrifuged (800 rpm, 4°C) for 5 min, and then serum-free DMEM/F12 was added to a final cell concentration of 1×10^7^cells/ml. The mice in the corresponding groups were then subcutaneously injected into the right back with 0.2 ml of cell suspension containing 2×10^6^ PER1-shRNA-I or Control-shRNA cells. Three weeks later, noticeable tumors were present, and the mice were sacrificed by cervical dislocation. The tumors were immediately excised, washed with TBS, dried on filter paper and weighed using a precise balance (AA250, Denver Instrument, USA). Tumor size was measured using a caliper, and tumor volume was calculated using the formula V=0.5×a×b^2^, where a is the longest diameter and b is the shortest diameter. The tumors were then divided equally into three parts. The first part was fixed in 4% paraformaldehyde, embedded in paraffin blocks, and cut into 4-μm slices. Routine HE staining was then performed, and the sections were observed under an optical microscope (200×). The second part was analyzed by flow cytometry as described above to measure cell proliferation and apoptosis after tissue homogenization. The third part was tested by qRT-PCR as mentioned above to detect mRNA expression of CLOCK, BMAL1, PER1, PER2, PER3, DEC1, DEC2, CRY1, CRY2, TIM, CKIε, RORα, NPAS2 and REV-ERBα after tissue homogenization. This experiment was conducted in strict accordance with the recommendations of the Guide for the Care and Use of Laboratory Animals of the Chongqing Medical University. All animal experimental protocols were approved by the Ethics Committee of Chongqing Medical University (Permit Number: CQMU 2011-28).

### Statistical analysis

All statistical analyses were performed using SPSS 19.0 software (IBM Corporation, Chicago, USA). Data are expressed as the mean ± SD. Comparisons between multiple groups were made using one-way analysis of variance (ANOVA). Comparisons between two groups were made using the least significant difference (LSD) test. Student's t-test was used to analyze differences between two groups of tumorigenesis assay. Values of *P<0.05* were considered to be statistically significant.

## SUPPLEMENTARY FIGURE AND TABLE


