# Supplementary material for: The clock gene PER1 plays an important role in regulating the clock gene network in human oral squamous cell carcinoma cells
Source: Oncotarget. 2016 Sep 2;7(43):70290–302. doi: 10.18632/oncotarget.11844 (PMC5342553; doi:10.18632/oncotarget.11844)
Supplement: Supplementary file 1 [file oncotarget-07-70290-s001.pdf]

The clock gene *PER1* plays an important role in regulating the clock gene network in human oral squamous cell carcinoma cells

SUPPLEMENTARY FIGURE AND TABLE

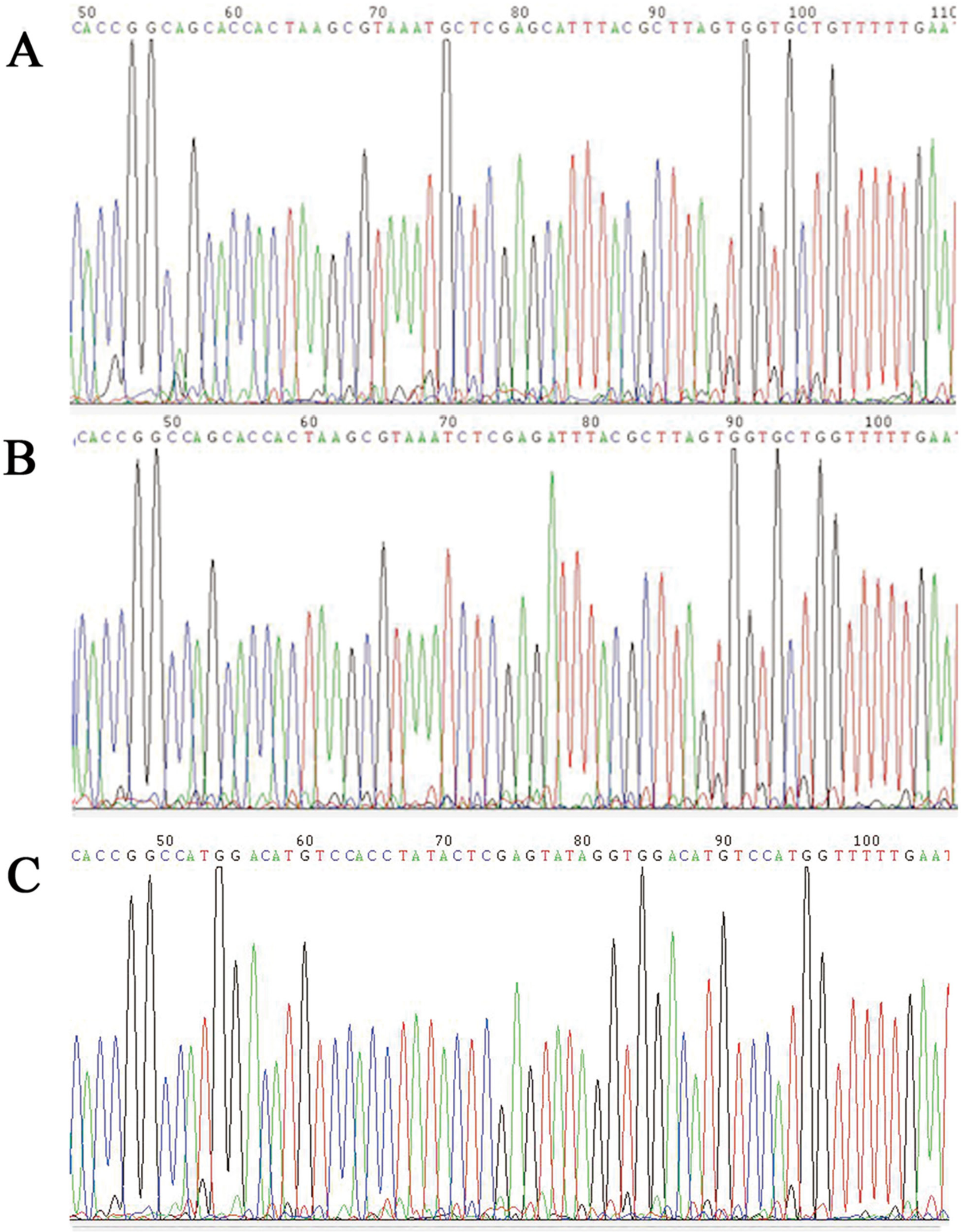

Supplementary Figure S1: DNA sequencing results of lentivirus PER1-shRNA plasmids. A. PER1-shRNA-I; **B.** PER1-shRNA-II; **C.** PER1-shRNA-III.

Supplementary Table S1: Sequences of PER1-shRNA interference

Group	Sense strand
PER1-shRNA-I	5’-CCGGCAGCACCACTAAGCGTAAATGCTCGAGCATTTACGCTTAGTGGTGCTGTTTTTG-3’
PER1-shRNA-II	5’-CCGGCCAGCACCACTAAGCGTAAATCTCGAGATTTACGCTTAGTGGTGCTGGTTTTTG-3’
PER1-shRNA-III	5’-CCGGCCATGGACATGTCCACCTATACTCGAGTATAGGTGGACATGTCCATGGTTTTTG-3’
